# Accelerations of the Fetal Heart Rate in the Screening for Fetal Growth Restriction at 34-38 Week’s Gestation

**Published:** 2021-10-30

**Authors:** HJ Odendaal, IC Crockart, C Du Plessis, L Brink, CA Groenewald

**Affiliations:** 1 Department of Obstetrics and Gynaecology, Stellenbosch University, South Africa; 2 Department of Mechanical and Mechatronic Engineering, Stellenbosch University, South Africa

**Keywords:** Machine learning, Umbilical artery, Doppler velocimetry, Fetal heart rate, Fetal heart rate accelerations, Fetal movements

## Abstract

**Objectives:**

To use machine learning to determine what information on Doppler velocimetry and maternal and fetal heart rates, collected at 20-24 weeks gestation, correlates best with fetal growth restriction according to the estimated fetal weight at 34-38 weeks.

**Study design:**

Data of 4496 pregnant women, collected prospectively for the Safe Passage Study, from August 2007 to August 2016, were used for the present analysis. Doppler flow velocity of the uterine, umbilical, and middle cerebral arteries and transabdominally recorded maternal and fetal ECGs were collected at 20-24 weeks gestation and fetal biometry collected at 34-38 weeks from which the estimated fetal weight was calculated. Fetal growth restriction was defined as an estimated fetal weight below the 10th centile. Accelerations and decelerations of the fetal and maternal heart rates were quantified as gained or lost beats per hour of recording respectively. Machine learning with receiver operative characteristic curves were then used to determine which model gives the best performance.

**Results:**

The final model performed exceptionally well across all evaluation metrics, particularly so for the Stochastic Gradient Descent method: achieving a 93% average for Classification Accuracy, Recall, Precision and F1-Score to identify the fetus with an estimated weight below the 10th percentile at 34-38 weeks. Ranking determined that the most important standard feature was the umbilical artery pulsatility index. However, the excellent overall accuracy is likely due to the value added by the pre-processed features regarding fetal gained beats and accelerations.

**Conclusion:**

Fetal movements, as characterized by gained beats as early as 20-24 weeks gestation, contribute to the value of the flow velocimetry of the umbilical artery at 34-38 weeks in identifying the growth restricted fetus.

## Introduction

Prevention of stillbirths is a high priority [1-3]. Placental pathology is closely associated with fetal growth restriction [4], for which small-for-gestational-age is commonly used as a proxy. Although fetal growth restriction is one of the most common complications of pregnancy [5], the majority of small-for-gestational-age babies are not recognized before birth [6]. Doppler flow velocimetry is one of the key investigations in the management of suspected intrauterine growth restriction (IUGR) [7]. However, there is still a need for improvement. In a recent study, a significant association was found between abnormal Doppler results and asymmetric fetal growth, but the sensitivity was only 3.9% [8]. To improve the sensitivity of screening, a combination of methods has recently been introduced, for example, the addition of a combination of novel biomarkers alongside ultrasound [9], or the integration of Doppler velocimetry with biophysical profiling [7]. A limitation of the abovementioned approaches is that they are hypotheses driven. As the generation of hypotheses is restricted to one’s knowledge of the relevant conditions; possibilities beyond one’s knowledge are therefore not considered and, as such, not investigated. In this regard, machine learning may be valuable to investigate possibilities beyond the traditional methodology. In the past decade, there has been a considerable increase in the investigation of the role of machine learning in medicine [10-12]. Machine learning presents a unique opportunity to find patterns and predictive value in the information already available, offering novel insight into old problems. Supervised learning in particular, with its use of test and training data, offers great potential in its ability to apply patterns to never-before-seen data [13]. Fetal heart rate (FHR) accelerations are synchronized with fetal movement bursts [14]. For this study we will quantify fetal movements by using FHR accelerations as a proxy reflected by the number of gained beats per hour [15], based on the original work on the non-stress test [16]. Prospectively collected data on fetal physiology for the Safe Passage Study (SPS) [17], will be investigated via supervised learning techniques to assess which fetal information collected at 20-24 weeks’ gestation (actually 20+0 to 23+6) correlates best with an estimated fetal weight (EFW) <10th percentile at 34-38 weeks (actually 34+0 to 37+6).

## Methods

**Figure 1:**
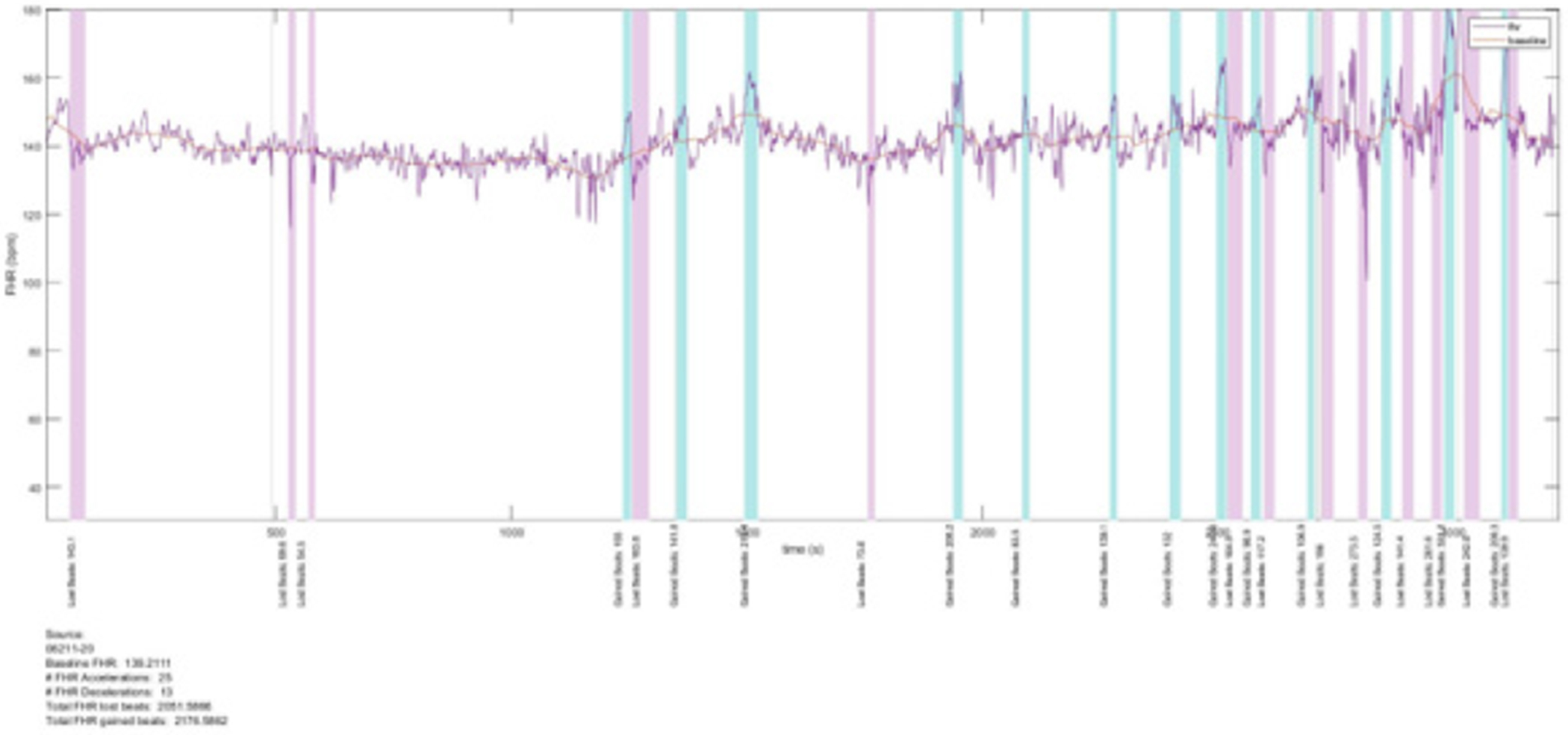
An example graph showing the gained and lost beats of the fetal heart rate at 20 to 24 weeks.

Recruitment for the prospective Safe Passage Study (SPS) was done over 7 and a half years [17]. An essential part of the fetal assessment was the non-invasive transabdominal recording of the maternal and fetal electrocardiograms (fECG, mECG) and the performance of an ultrasound examination for Doppler flow velocity waveforms and fetal biometry at 20-24 weeks and 34 to 38 weeks gestation. The ECGs were recorded with the AN24 Fetal Halter device (Monica Health Care, Nottingham, UK). It has previously been demonstrated that this device is extremely successful to record the fECGs at 20 to 24 weeks gestation at a success rate of 95.4% [18]. Dedicated ultrasonologists performed all the ultrasound examinations according to a strict protocol [19]. Fetal biometry included head circumference, biparietal diameter, abdominal circumference and femur length. Hadlock’s formula was used to calculate the estimated fetal weight (EFW) from the biometric measurements [20]. We also added the mECG, as we previously found in a subgroup of pregnant women at term that uterine activity was associated with periodic decelerations of the maternal heartrate (MHR) [21]. The raw signals of the mECG and fECG were then processed to determine the various heart rates and the lost and gained beats for the maternal and fetal heart rates. The acceleration area above the basal heart rates and the deceleration area below the basal heart rates were then calculated as gained or lost beats respectively and expressed as per hour as the durations of the recordings varied (Figure 1) [15].

The EFW at the 34-38 weeks assessments were compared with the growth curves of Hadlock to determine whether the EFW was below the 3rd or 10th percentiles [20]. As the methods of machine learning have been published in detail [22], we only provide a summary. Once the raw data had been processed and the metrics prepared, the master data set was split into two main datasets: one for the information relevant to the gestation period including relevant information for both mother and fetus; the other contained all the processed data for the gained/lost beats of both mother and fetus (Table 1).

The two datasets were then analysed and prepared separately, which allowed one to assess the data quality of each. Before the application of supervised learning techniques, the data was correctly prepared using the CRISP-DM methodology [22]. Following standard procedures, the data was firstly analysed, and data quality reports were produced. These allowed a succinct feature-by-feature breakdown of the quality of the data. These reports provided information regarding how much of the data was missing, how many unique values were present and the distribution of the data. After the quality had been assessed, the relationships between the features were visualised using density graphs. The two datasets were then merged to allow direct comparison between features of each set. The overall shapes of the graphs were then recorded, each shape requiring a different correction procedure. An analysis of the correlation and covariance of the data followed, where scatter plot facet grids were used to compare the relationship between sets of features. A data quality plan was then finalised, which detailed the findings of the analysis phase and contained potentially viable strategies to mitigate the identified issues. Once analysed, the data was prepared for use in a predictive model. This process included cleaning the data, removing unnecessary features, simplifying the final dataset and transforming the dataset for the modelling phase. Included in this phase was the application of sampling, to balance the target attribute’s positive/negative ratio, and the normalization of the remaining data. The features were then ranked in order of Information Gain (a metric calculated by the Orange software [23]) and a tree model was produced. The combination of these two models informed the decision to construct a feature that would improve the predictive success of the model. Finally, the features for the final model were selected, simplifying the dataset to 5 features, 2 constructed features, the target variable and a meta-attribute (the unique row ID).

Several baseline models were created at critical stages, each allowed a separate method to be explored to improve the success of the final model. The modelling process made use of several algorithms and methods to assess the predictive value of the data. The following methods were used: Gradient Descent (SGD) & Regularization, Logistic Regression & Classification, Nearest Neighbors Method, and Random Forest.

The methods above were applied after clustering had been applied to some models. The collection of models was narrowed down to 6 of the most successful. For the evaluation of the predictive model several metrics were used to represent the different aspects of the model’s performance. These included the area under curve (AUC) values, classification accuracy (CA), an F-1 Score, Precision and Recall [24]. The test data set was compared to the data used for training to evaluate the model as a learning algorithm, ensuring its performance is evaluated using information not yet seen. Random sampling was used, repeated 10 times per sample, with a training set size of 70%. The evaluation was further bolstered by stratification. The results were then averaged over both target attribute classes, namely ‘1’ and ‘0’ [25]. Machine learning was then used to determine which maternal and fetal data collected at 20 to 24 weeks and Doppler data collected at 34-38 week’s gestation correlated best with the diagnosis of IUGR at 34-38 weeks. Various models were evaluated but it was shown that Model 5 and 6 had the largest AUC values [25]. The results of these models were then used for further analyses.

## Results

There were 4764 pregnant women in the study (Table 1).

The mean maternal age and BMI was 24.6 years and 25.5 kg/m2 respectively. Participants enrolled at around 22.6 and 35.1 weeks for the 20-24 and 34-38 week assessments respectively. The mean gestational age at delivery was 38.7 weeks, the mean birthweight 3002.9 g, and the mean birthweight Z score −0.38 (Table 2).

**Figure 2:**
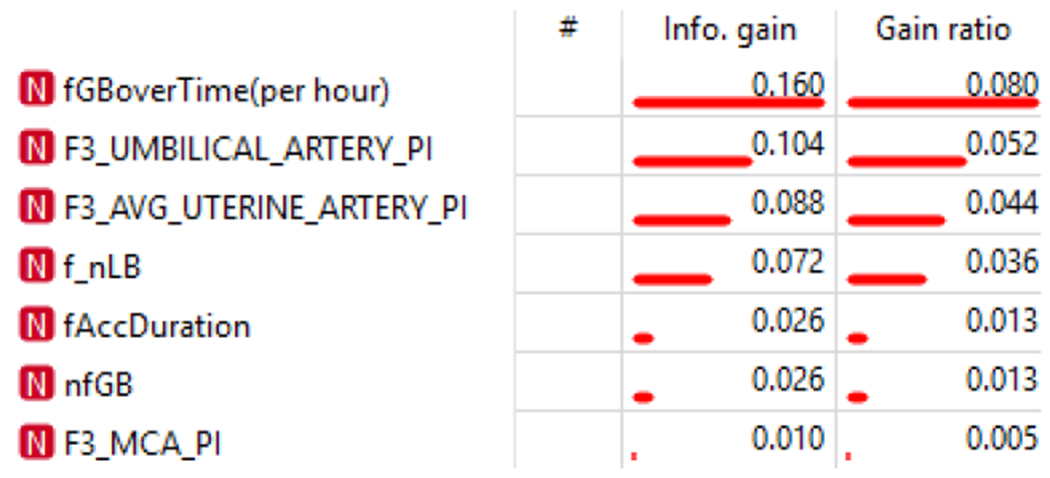
Feature ranking in terms of Information Gain, a metric determined by the Orange software. fGB=fetal gained beats, F3_UMBILICAL_ARTERY_PI= Mean pulsatility index of the umbilical artery at 34-38 weeks, F3_AVG_UTERINE_ARTERY_PI=Mean pulsatility index of the uterine artery at 34-38 weeks, f_nLB=number of fetal lost beats, fAccDuration=Duration of fetal heart rate acceleration; nfGB=number of fetal gained beats, F3_MCA_PI= Mean pulsatility index of the middle cerebral artery at 34-38 weeks.

The most common complications of pregnancy encountered were preeclampsia (212; 3.8%), stillbirth (82; 1.4%) and placental abruption (49; 0.9%). The minority of infants were male (2,867; 49.3%). According to the estimated fetal weight at 34-38 weeks, 17 (2.1%) of fetuses weighed less than the 3rd centile and 40 (5.0%) less than the 10th centile. The final model performed exceptionally well across all evaluation metrics, particularly so for the Stochastic Gradient Descent method: achieving a 93% average for Classification Accuracy, Recall, Precision and F1-Score to identify the fetus with an estimated weight below the 10th percentile at 34-38 weeks. This accuracy is likely due to the value added by the pre-processed features regarding fetal gained beats and accelerations. A full technical breakdown of the results can be found in the technical article [25]. During the process, a feature ranking table was produced (Figure 2), ranking the relevant features in terms of their Information Gain (metric for correlation between presence of feature and successful classification). The ranking table expressed that the most important standard feature was the umbilical artery pulsatility index, in accordance with the literature. Additionally, scoring even better than the standard features, the processed feature for the ratio of fetal gained beats over time (per hour) ranked as most informative. According to the Confusion Matrix produced [23], true positive and true negative findings were seen in 66.7% to 93.5% of analyses, in contrast to 6.5% to 33.3% of false negative findings, a vital part of the review of the model, since a model can perform well in terms of classification accuracy but still have very skewed results in terms of true/false positive/negatives. A Confusion Matrix is especially crucial when dealing with smaller sample sizes as it ensures that the performance success is not simply due to a skewed set of positive vs negative samples.

**Figure 3:**
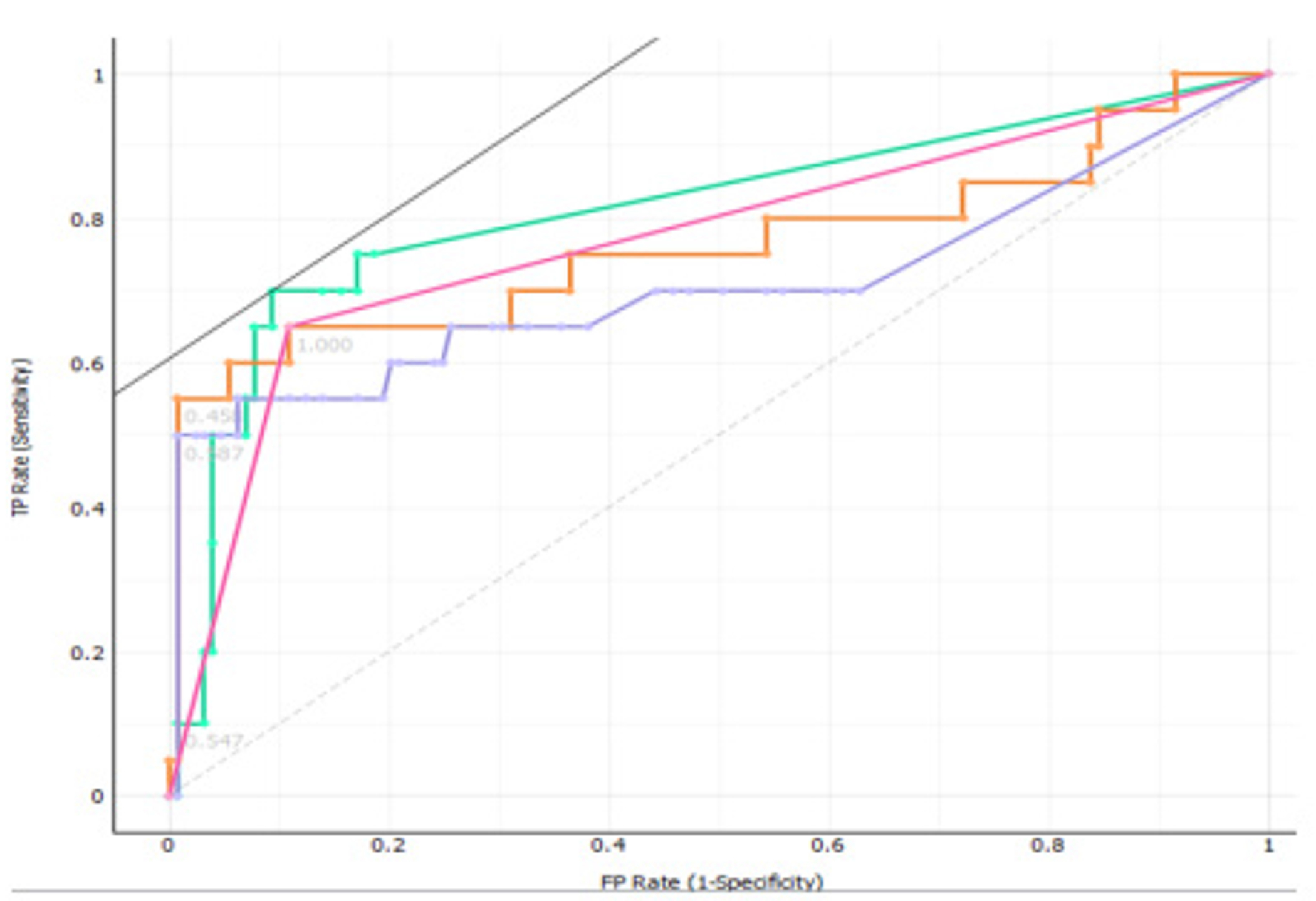
The receiver operator characteristic curve analysis graph for the four algorithms used.

The Receiver operating characteristic (ROC) curve analysis, exploring the ratio between the true positive and the false positive values, demonstrated an area under curve of 0.771, 0.707, 0.762 and 0.812 for Stochastic Gradient Descent, Random Forest, Logistic Regression and K-Nearest Neighbors respectively (Figure 3).

Using machine learning, we have confirmed the value of the pulsatility index (PI) of the umbilical artery Doppler flow velocity waveform at 34-38 weeks gestation to identify the pregnant women at risk for fetal growth restriction [26]. However, found that the predictive value of a raised PI could be improved by also taking into consideration fetal gained beats, reflecting fetal heart rate accelerations or movements at 20-24 weeks. Doppler velocimetry followed by appropriate intervention could reduce perinatal mortality by as much as 29% [27]. However, there is still a need for more accurate identification of risks of fetal growth restriction at earlier gestational ages. In this regard, fetal biometry combined with maternal serum biomarkers seems to be promising [9]. Another approach is to combine Doppler flow velocity with fetal behaviour as reflected by reduced fetal movements in response to a deficient intrauterine environment. Pregnancies complicated by multiple episodes of reduced fetal movements show significant changes in the PI of the middle cerebral artery. This is likely to be due to worsening fetal hypoxemia in women presenting with recurrent reduced fetal movements [28]. Women perceive between 2.4% and 81.0% (median 44.8%) of fetal movements observed by ultrasound [29]. This low perception and the lack of appropriate clinical trials [30], are probably some of the reasons why the count of fetal movements are not recommended for use in clinical practice [31]. However, accelerations of the fetal heart rate can be used as a proxy for fetal movements. The large majority of FHR accelerations are associated with fetal movements, 95.5% during uterine contractions, and 90.9% when the uterus is relaxing [32]. The small accelerations are associated with 82.4% of fetal movements noticed by ultrasonography. The reassuring effects of the presence of accelerations [33], was one of the main reasons for the replacement of the oxytocin challenge test with the nonstress test [34]. Although a reduction in perceived fetal movements in women with fetal growth restriction, may lead to the consideration of expedited delivery, there has been little progress to objectively evaluate fetal movements for clinical use. Therefore, there are several requests for the better quantification of fetal movements [35-37]. SYSTEM 8000 was one of the first computerized interpretations of antenatal fetal heart rate pattern. Decelerations and accelerations were also quantified [16]. As fetal growth restriction (FGR) is associated with decreased movements in the absence of hypoxia, there is a great need to quantify it further and to use quantification of movements by looking at the deceleration area of the FHR [38]. In order to improve the accuracy of diagnosis if FGR is suspected there seems to be a tendency to combine biophysical and biochemical data [39,40]. In addition, the use of artificial intelligence seems to correctly select a panel of metabolites in cord blood serum to detect IUGR in the new-born [41]. As longitudinal assessment of fetal growth from the second to third trimester has a low predictive capacity for SGA and late FGR in low-risk singleton pregnancy, compared with cross-sectional growth evaluation [42], there is an urgent need for new technologies, or better application of existing ones, to objectively assess fetal movements in the low-risk setting and to characterize how these may relate to fetal health. In doing so, it may become possible for us to improve management of FGR, more precisely determine optimal delivery timing and potentially reduce stillbirths [43]. We found that fetal gained beats at 20-24 weeks improved on the value of the Doppler velocimetry to identify the growth restricted fetus. However, looking at gained beats in later pregnancy may further improve the value as fetal heart rate decelerations in later pregnancy when there is an increase in the number of accelerations [44].

## Conclusion

The key role of Doppler velocimetry in identifying the growth restricted fetus has been confirmed by machine learning. Additionally, the performance of the model has been improved by using fetal heart rate accelerations, expressed as gained beats per hour as a proxy for fetal movements.

## Figures and Tables

**Table 1:** The Feature Overview for each dataset, with a brief description of each feature. The names of the features have been changed for coherency purposes.

Fetal and maternal heart rate information collected at 20-24 weeks (n=4764)
The number of times gained beats were recorded for the maternal heart rate
The sum of all the gained beats recorded for the maternal heart rate
Ratio of the total gained beats recorded per hour of recording for the maternal heart rate
The total time (in seconds) of the entire recording that were recorded as accelerations for the maternal heart rate
The total time (in seconds) of the recording for the maternal heart rate
The number of times lost beats were recorded for the maternal heart rate
The sum of all the lost beats recorded for the maternal heart rate
The number of times gained beats were recorded for the fetal heart rate
The sum of all the gained beats recorded for the fetal heart rate
Ratio of the total gained beats recorded per hour of recording for the fetal heart rate
The total time (in seconds) of the entire recording that were recorded as accelerations for the fetal heart rate
The total time (in seconds) of the recording for the fetal heart rate
The number of times lost beats were recorded for the fetal heart rate
The sum of all the lost beats recorded for the fetal heart rate
**Doppler and Estimated fetal growth information collected at 34-38 weeks**
Pulsatility index for the umbilical artery (n=644)
Pulsatility index for the uterine artery (n=657)
Pulsatility index for the middle cerebral artery (n=638)
Whether or not the intrauterine growth restriction was less than 3% (n=429)
Whether or not the intrauterine growth restriction was less than 10% (n=429)

**Table 2:** Background maternal and fetal information of the participants.

Variable	Mean	SD	Median	Range
Maternal age (years)	24.6	5.9	24	16 - 45
Body mass index (kg/m^2^)	25.5	5.7	24.2	13.7 - 55.9
Gestation at enrolment (days)	134.8 (20.3 weeks)	46.4	132	38 - 272
Gestation at 20-24 weeks visit	158.5 (22.6 weeks)	6.3	160	136 −182
Gestation at 34-38 weeks visit	245.4 (35.1 weeks)	7.2	243	226 −270
Gestation at birth (days)	270.7 (38.7 weeks)	21.8	275	61 - 313
Birthweight (gram)	3002.9	583.7	3020	190 - 5740
Birthweight z-score	−0.38	1	−0.4	−5.7 - 4.1
